# Mediators of gender effects on depression among cardiovascular disease patients in Palestine

**DOI:** 10.1186/s12888-019-2267-4

**Published:** 2019-09-12

**Authors:** Hala Allabadi, Nicole Probst-Hensch, Abdulsalam Alkaiyat, Saleem Haj-Yahia, Christian Schindler, Marek Kwiatkowski, Elisabeth Zemp

**Affiliations:** 1 Department of Epidemiology and Public Health, Swiss Tropical and Public Health Institute, Socinstrasse 57, P.O. Box, 4002, Basel, Switzerland; 2 University of Basel, Petersplatz 1, 4001 Basel, Switzerland; 3 Faculty of Medicine and Health Sciences, An-Najah National University, Rafidia Street, P.O. Box 7, Nablus, Palestine; 4 An-Najah National University Hospital, Asira Street, Nablus, Palestine; 5 School of Clinical Sciences, University of Bristol, 69 St Michael’s Hill, Bristol, BS2 8DZ UK; 6 Institute of Cardiovascular and Medical Sciences, Glasgow University, 126 University Place, Glasgow, G12 8TA UK

**Keywords:** Gender, Depression, Cardiovascular disease, Mediators, Post-traumatic stress disorder

## Abstract

**Background:**

Among patients suffering from coronary heart disease (CHD) and comorbid depression, women experience a higher burden compared to men. Little is known on the characteristics that differentiate men and women with both diseases and whether these factors mediate gender effects on depression. This study assessed whether women are more likely to suffer from depression and which characteristics mediate gender effects on depression among a cardiac population in Palestine, specifically addressing the role of post-traumatic stress disorder (PTSD).

**Methods:**

Using a cross-sectional design, patients consecutively admitted with a CHD to one of the four main hospitals in Nablus, Palestine, were interviewed using a structured questionnaire with validated instruments. Data was also obtained from hospital medical records. Patients were assessed for depression using the Cardiac Depression Scale (CDS). Bivariate analysis was conducted to compare characteristics of women and men with and without depressive symptoms. Mediators (direct and indirect effects) of the association between gender and depression were evaluated using a structural equation model (SEM).

**Results:**

Women were more likely to suffer from severe depression than men (28.7% vs. 18.8%). Female gender was positively associated with higher PTSD symptoms, comorbidities, somatic symptoms and income, and with lower resilience, self-esteem, quality of life, education, prevalence of smoking and physical activity. Structural equation modeling revealed negative indirect effects of gender on depression (CDS score) through resilience, self-esteem and physical activity, whereas positive indirect effects of gender on depression were observed through PTSD, comorbidities, somatic symptoms and smoking. There was no direct effect of gender on depression.

**Conclusion:**

This study found a higher prevalence of severe depression in female patients with cardiac disease compared to male cardiac patients. Our findings provide novel information on mediating factors of the association between gender and depression among cardiac patients, in particular PTSD. The results emphasize the need for further research on potential mediating factors that could account for gender differences in depression and the need to provide support programs for female patients with comorbid CHD and depression to improve their psycho-social well-being.

**Electronic supplementary material:**

The online version of this article (10.1186/s12888-019-2267-4) contains supplementary material, which is available to authorized users.

## Introduction

Depression is an increasingly recognized risk factor for cardiovascular disease (CVD), particularly coronary heart disease (CHD) [1, 2]. The prevalence of depression among cardiac patients ranges from 15 to 30%, which is considerably higher than in the general population [3]. Depression in cardiac patients is associated with poor outcomes [4], long-term prognosis [5], overall quality of life [6], mortality [1], and a dose-response relationship has been reported between depressive symptom level and subsequent mortality [7].

Depression is up to two times higher in women than in men [8, 9]. Many factors could explain these differences including biological, genetic, psychological and social differences between women and men [10]. Gender and sex are often used interchangeably suggesting biological and psychosocial attributes vary with one another [11]. While sex denotes genetic and biological characteristics, gender refers to the array of socially constructed roles and relationships, personality traits, attitudes, behaviors and values that society ascribes to the sexes on a differential basis [12]. However, the use of the term sex as a ‘stand-alone indicator of biology’ is rejected as gendered experiences materialize in the body and measures of sex can include effects of gender [13]. In the case of depression and in the presence of sex differences, biology alone cannot provide an accurate explanation. Higher depression rates are found in women than men due to stressors related to social roles whereas lower rates of depression are found among men because male-attributed symptoms of depression such as anger, aggression, irritation, and abusive behavior are commonly not recognized as depressive symptoms [14]. Evidence suggests that women underprivileged in different aspects of life suffer from greater stressors and poorer health than men, including mental disorders and chronic diseases [15]. Women also report poorer health than men due to lack of access to healthcare and due to the stressors caused by their gender and marital roles [16, 17].

The role of gender for CHD has been addressed extensively in literature since the 1980’s [6, 18]. Studies have reported that being female is associated with higher mortality and morbidity after cardiac events compared to being male [19, 20] and that women are likely to develop more depressive symptoms associated with CHD than men [21]. A meta-analysis found an overall prevalence of comorbid major depression of 18.7% in women compared to 12.0% in men. In particular, women are more vulnerable after coronary artery bypass graft (CABG) compared to men, suffer from more comorbid illness [22], are more likely to experience anxiety and more likely to be socially isolated [23]. Among myocardial infarction (MI) patients, women report less social support and more role disruption during recovery [24] and are two times more prone to depression post-MI than men [25]. Therefore, female cardiac patients have a higher risk of suffering from adverse effects of depression on the prognosis of their disease [1, 9, 26]. In addition, women have greater symptom frequency, experience greater risk of re-infarction, have lower survival rates after CABG and higher rates of subsequent heart failure (HF) compared to men [27–29]. In several studies, the worse cardiac outcomes in women persist after adjustment for age, medical history, clinical severity, hospital treatment, and cardiac procedures [28, 30–32]. Despite the burden observed in women due to comorbid CHD and depression, they continue to be understudied in research and data are often not presented by gender. The high burden of CVD and the rise of depression rates among women in the general population as well as in CHD populations call for assessment of the gender gap in depression. There is scarce literature on characteristics that distinguish men and women with comorbid CHD and depression. Furthermore, whether these factors mediate gender effects on depression in cardiac patients remains unclear. This ultimately makes it difficult to identify women at higher risk of adverse outcomes associated with both CHD and depression. In this paper, the terms sex and gender as well as women and men are used: ‘gender’ as an overarching notion; ‘sex’ when referring to associations reported in the literature for being female and male respectively; and the terms ‘women’ and ‘men’ to denote population subgroups.

Post-traumatic stress disorder (PTSD) is also a common co-morbid condition in cardiac patients [33] and rates are higher among female cardiac patients. PTSD has been associated with recurrent MI, hospitalizations and mortality [34, 35]. Similarly, PTSD exhibits gender-specific patterns and is known to be comorbid with depression [36].

Previous literature has focused on gender differences in depression among heart disease patients mainly in high income countries [18, 27]. In Middle Eastern countries, CVD accounts for 34% of all deaths and PTSD is high among individuals living in regions of conflict [37]. The Occupied Palestinian Territories (oPt) in particular, report high rates of depression in response to the stressful living conditions. The different societal roles of women and the evidence based CVD-gender differences call for more research into the understanding of gender-related factors with regard to depression and mediating factors among cardiac patients. In this study, we sought to assess whether Palestinian women are more likely to suffer from depression than Palestinian men among a sample of 1022 consecutively admitted cardiac patients at one of the four major hospitals in the West Bank city of Nablus, Palestine, and to investigate whether socio-demographic, clinical, psychosocial and lifestyle characteristics mediate gender effects on depression, especially addressing the role of PTSD.

## Methods

### Study design and population

This cross-sectional hospital-based study was conducted on consecutive patients aged 30–80 years admitted to the cardiology and cardiac surgery departments at one of the four main hospitals in Nablus, Palestine, during their stay in the hospital (1–7 days) between March 2017 and November 2017. They were included in the study if they were diagnosed with CHD, ST elevation or non-ST elevation MI, angina, HF, cardiac arrhythmia, valve disease or any other cardiac disease. Patients with a normal cardiac catheterization (CATH), an acute or past stroke, end-stage kidney disease (including dialysis patients), peripheral vascular disease, psychological disorders, cognitive deficit, neurological disorders (dementia, Alzheimer’s disease, epilepsy, Parkinson’s disease), use of antidepressant or any other condition affecting the quality of their responses were excluded from the study. A total of 1092 patients were approached and met initial eligibility requirements, and 1053 agreed to participate in the study. The analytic sample included 1022 subjects. The study was approved by the Ethics Committee of Nordwest-und Zentral Schweiz (EKNZ) in Basel, Switzerland, and by the Institutional Review Board (IRB) committee at An-Najah National University in Nablus, Palestine.

### Data collection

Eligible patients were approached for private in-person interviews using hospital registries and written informed consent was obtained from those who agreed to participate. Prior to completion of the interview, socio-demographic and clinical characteristics including: age, gender, marital status, residence, education level, occupation, income; current diagnosis, previous cardiac diagnoses, years with cardiac disease, cardiac treatment (at admission), co-morbidities, medications and family history of CVD were retrieved from patients’ medical records and were ascertained via patient interview. In-person interviews consisted of a sequence of validated instruments assessing physical and psychosocial factors and questions assessing lifestyle factors.

### Study measurements

Depressive symptoms were assessed by the Cardiac Depression Scale (CDS), a disease-specific, 26-item questionnaire used to accurately measure depression in patients with CVD. The CDS is scored based on a Likert scale ranging from strongly disagree (1) to strongly agree (7). Total scores are calculated as the sum of the scores of each item and range from 26 to 182 [38]. The CDS can be used as a continuous measure, where higher scores indicate more severe depressive symptoms, or as an ordinal indicator of possible depression using previously established cut-off points which include: < 90 for no depression; 90–100 for mild to moderate depression; and >100 for severe depression. These cut offs were used in the current study to categorize depressive symptoms. The CDS has a sensitivity of 88% and a specificity of 84% for severe depression when a cutoff of > 100 is used, and 84% sensitivity and 78% specificity for cutoff scores of 90–100 [39]. Further instruments used include the Post-Traumatic Stress Disorder Checklist (PTSD-PCL-S) [40]; Enhancing Recovery in Coronary Heart Social Support Instrument (ESSI) [41, 42]; Resilience Scale-14 (RS-14) [43]; Single-Item Self-Esteem Scale (SISE) [44]; Short-form 12 Health Survey (SF-12-PCS; SF-12-MCS) [45] and the Patient Health Questionnaire-15 (PHQ-15) [46]. The instruments have been described in detail elsewhere [47].

### Statistical analyses

Depressive symptoms were examined both as a continuous variable (CDS score) and as a dichotomous variable (presence of moderate-severe depressive symptoms using the standard cutoff of CDS ≥90). Characteristics of women and men with and without depressive symptoms were compared using Chi-squared or Fisher exact tests for categorical variables, and independent *t*-tests for continuous variables, as appropriate.

A structural equation model (SEM) was built to assess direct and indirect (i.e. mediated) effects of gender on depression. Mediators are variables which transfer part of the effect of an independent variable (IV) on a dependent variable (DV). In our analysis, the IV was gender (used as a binary variable, with “being female” coded as 1), the DV was depression (CDS score, used as a continuous variable), and the potential mediator variables were socio-demographic, clinical, psychosocial and lifestyle factors [48]. First, mediator groups (i.e. socio-demographic, clinical, psychosocial and lifestyle factors) were assessed in separate models. In a second step, the final model was built (reference model, *N* = 1022), including education, income, comorbidities, somatic symptoms, quality of life, PTSD, resilience, self-esteem, smoking, physical activity. These potential mediators were chosen based on 1) their statistical significance in the preceding analyses, using a cut-off level of *p* < 0.10 2) the magnitude of their association 3) whether the associations were significant between gender and the potential mediator, as well as between the potential mediator and depression (CDS score). The SEM was estimated using the maximum likelihood method and included continuous, binary and ordinal variables which were all treated as continuous. Inclusion of a larger number of potential mediators reduces bias in estimating the true direct effect of gender on depression (CDS score). Potential confounding factors assessed were age, marital status, occupation, social support, and lifestyle factors (i.e. BMI, alcohol consumption). A further SEM-analysis was then conducted stratifying by age, running separate models for two age groups (those aged below and above the median age of 59 years; see Additional file 1: Figure S1a and b). Moderation by age was assessed by testing the differences between corresponding age-specific effects using Chi-squared tests. In further analyses, two additional SEM’s were computed to assess whether resilience and smoking could be potential mediators and/or moderators of the effect of PTSD on depression, adding the respective factor as mediator between PTSD and depression and testing its interaction with PTSD to assess potential moderation. This additional analysis was based on general conceptualization and literature on psychological comorbidity of PTSD and depression [36, 49]. Analysis was conducted using the STATA Statistical Software Release 15 (StataCorp., College Station, U.S.A.).

## Results

This sample consisted of 750 (73.4%) men and 272 (26.6%) women. Among the cardiac patients with depressive symptoms, 231 (28.7%) were women. In contrast, among patients without depressive symptoms, 41 (18.8%) were women. Socio-demographic, clinical, psychosocial and lifestyle factors of depressed and non-depressed men and women are presented in Table 1. In terms of socio-demographic factors, among those with depressive symptoms, women were on average older than men, more likely to be unmarried (i.e. single, widowed), less educated, unemployed and have no income compared to men.

**Table 1:** Bivariate comparison of study characteristics in men and women with CVD, by presence and absence of depressive symptoms, *N* = 1022

Variable	Sample with depressive Symptoms (CDS ≥90) (*N* = 804)			Sample without depressive symptoms (CDS < 90) (*N* = 218)		
	Male (*N* = 573) *N* (%)	Female (*N* = 231) *N* (%)	*P* value	Male (*N* = 177) *N* (%)	Female (*N* = 41) *N* (%)	*P* value
Socio-demographic factors						
Age, mean (SD)^a^	57.9 ± 9.6	61.6 ± 10.5	**< 0.001**	57.8 ± 10.2	63.7 ± 10.7	**0.001**
Marital status			**< 0.001**			**< 0.001**
Married	564 (98.4)	171 (74.0)		166 (93.8)	25 (60.7)	
Not married	9 (1.6)	60 (26.0)		11 (6.2)	16 (39.0)	
Residence			0.456			0.723
City	261 (45.5)	104 (45.0)		89 (50.3)	20 (48.8)	
Village	270 (47.1)	104 (45.0)		79 (44.6)	20 (48.8)	
Camp	42 (7.3)	23 (10.0)		9 (5.1)	1 (2.4)	
Education degree			**< 0.001**			**0.004**
No HS diploma	298 (52.0)	188 (81.4)		84 (47.4)	30 (73.2)	
HS diploma	173 (30.2)	32 (13.8)		49 (27.7)	9 (21.9)	
College degree	102 (17.8)	11 (4.8)		44 (24.9)	2 (4.9)	
Occupation			**< 0.001**			**< 0.001**
Professional	153 (26.7)	9 (3.9)		47 (26.6)	0 (0.0)	
Non-professional	226 (39.4)	4 (1.7)		76 (42.9)	1 (2.4)	
Unemployed	143 (25.0)	175 (75.8)		31 (17.5)	29 (70.8)	
Retired	51 (8.9)	4 (1.7)		23 (13.0)	3 (7.3)	
House wife	0 (0.0)	39 (16.9)		0 (0.0)	8 (19.5)	
Income			**< 0.001**			**< 0.001**
Yes	442 (77.1)	77 (33.3)		146 (82.5)	12 (29.3)	
No	131 (22.9)	154 (66.7)		31 (17.5)	29 (70.7)	
Clinical Factors						
Cardiac diagnosis			**< 0.001**			**0.010**
CHD	188 (32.8)	63 (27.3)		70 (39.6)	13 (31.7)	
MI	242 (42.2)	76 (32.9)		73 (41.2)	15 (36.6)	
Angina	96 (16.8)	38 (16.4)		24 (13.6)	4 (9.8)	
Other	47 (8.2)	54 (23.4)		10 (5.6)	9 (21.9)	
Previous cardiac diagnosis			**0.036**			0.898
Yes	386 (67.4)	173 (74.9)		106 (59.9)	25 (61.0)	
No	187 (32.6)	58 (25.1)		71 (40.1)	16 (39.0)	
Years with cardiac disease			0.356			0.800
≤1 year	347 (60.6)	130 (56.3)		124 (70.1)	27 (65.8)	
2–9 years	148 (25.8)	61 (26.4)		37 (20.9)	9 (22.0)	
≥ 10 years	78 (13.6)	40 (17.3)		16 (9.0)	5 (12.2)	
Cardiac treatment (at admission)			**0.005**			0.872
CATH/stent	306 (53.4)	98 (42.4)		107 (60.5)	23 (56.0)	
CATH/CABG	139 (24.3)	58 (25.1)		34 (19.2)	9 (22.0)	
CATH/other & unknown	128 (22.3)	75 (32.5)		36 (20.3)	9 (22.0)	
Co-morbidities			**< 0.001**			**0.021**
None	182 (31.8)	32 (13.9)		73 (41.2)	12 (29.3)	
1	169 (29.5)	68 (29.4)		57 (32.2)	9 (22.0)	
2+	222 (38.7)	131 (56.7)		47 (26.6)	20 (48.7)	
Medications			**0.003**			0.471
None	81 (14.1)	13 (5.6)		32 (18.1)	6 (14.6)	
1–2	85 (14.9)	37 (16.0)		22 (12.4)	8 (19.5)	
3–4	407 (71.0)	181 (78.4)		123 (69.5)	27 (65.8)	
Somatic symptoms (PHQ-15)			**< 0.001**			**0.046**
Minimal	48 (8.4)	6 (2.6)		35 (19.8)	2 (4.9)	
Low	118 (20.6)	34 (14.7)		74 (41.8)	15 (36.6)	
Medium	193 (33.7)	64 (27.7)		43 (24.3)	14 (34.1)	
High	214 (37.3)	127 (55.0)		25 (14.1)	10 (24.4)	
Family history			0.356			0.033
Yes	236 (41.2)	87 (37.7)		63 (35.6)	22 (53.7)	
No	337 (58.8)	144 (62.3)		114 (64.4)	19 (46.3)	
QoL, (SF-12-PCS score), mean (SD)^a^	38.2 ± 12.1	31.4 ± 10.1	**< 0.001**	44.6 ± 12.1	34.4 ± 1.9	**< 0.001**
Psychosocial factors						
PTSD (PTSD-PCL-S)			**< 0.001**			0.282
Minimal	329 (57.4)	105 (45.4)		147 (83.1)	30 (73.2)	
Some	66 (11.5)	15 (6.5)		11 (6.2)	3 (7.3)	
Moderate	141 (24.6)	85 (36.8)		19 (10.7)	8 (19.5)	
High	37 (6.5)	26 (11.3)		0 (0.0)	0 (0.0)	
Social support (ESSI)			**< 0.001**			0.293
Low	186 (32.5)	108 (46.7)		54 (30.5)	16 (39.0)	
High	387 (67.5)	123 (53.3)		123 (69.5)	25 (61.0)	
Resilience (RS-14)			**< 0.001**			**0.002**
Very low	45 (7.9)	38 (16.4)		3 (1.7)	6 (14.6)	
Low	55 (9.6)	38 (16.4)		8 (4.5)	2 (4.9)	
Low-end	118 (20.6)	46 (19.9)		37 (20.9)	3 (7.3)	
Moderate	148 (25.8)	52 (22.5)		45 (25.4)	12 (29.3)	
Moderately-high	156 (27.2)	45 (19.5)		58 (32.8)	15 (36.6)	
High	51 (8.9)	12 (5.1)		26 (14.7)	3 (7.3)	
Self-esteem (SISE), mean (SD)^a^	5.9 ± 1.3	5.2 ± 1.6	**< 0.001**	6.2 ± 1.0	6.1 ± 1.2	0.633
QoL,(SF-12-MCS score), mean (SD)^a^	39.1 ± 12.5	35.3 ± 3.3	**< 0.001**	46.2 ± 12.6	47.0 ± 1.9	0.724
Lifestyle factors						
Smoking status			**< 0.001**			**< 0.001**
Never	106 (18.5)	182 (78.8)		42 (23.7)	34 (82.9)	
Former	126 (22.0)	11 (4.8)		105 (59.3)	4 (9.8)	
Current	341 (59.5)	38 (16.4)		30 (17.0)	3 (7.3)	
Currently on diet			0.767			0.483
Yes	87 (15.2)	37 (16.0)		39 (22.0)	7 (17.1)	
No	486 (84.8)	194 (84.0)		138 (78.0)	34 (82.9)	
Fat consumption			**< 0.001**			**0.075**
Low	247 (43.1)	127 (55.0)		85 (48.0)	21 (51.2)	
Medium	176 (30.7)	77 (33.3)		53 (30.0)	17 (41.5)	
High	150 (26.2)	27 (11.7)		39 (22.0)	3 (7.3)	
Vegetable & fruit consumption			0.920			0.789
Low	59 (10.3)	25 (10.8)		15 (8.5)	3 (7.3)	
Medium	200 (34.9)	83 (35.9)		55 (31.1)	15 (36.6)	
High	314 (54.8)	123 (53.3)		107 (60.4)	23 (56.1)	
Alcohol use			**0.004**			0.100
Yes	35 (6.1)	3 (1.3)		11 (6.2)	0 (0.0)	
No	536 (93.9)	228 (98.7)		165 (93.8)	41 (100.0)	
Physical activity			**< 0.001**			**0.008**
None	178 (31.1)	117 (50.7)		31 (17.5)	11 (26.8)	
Not daily	116 (20.2)	37 (16.0)		24 (13.6)	12 (29.3)	
Daily	279 (48.7)	77 (33.3)		122 (68.9)	18 (43.9)	
BMI			**0.083**			0.230
Underweight	3 (0.5)	0 (0.0)		1 (0.6)	0 (0.0)	
Normal weight	118 (20.6)	39 (16.9)		38 (21.5)	4 (9.8)	
Overweight	236 (41.2)	84 (36.4)		87 (49.1)	20 (48.8)	
Obese	216 (37.7)	108 (46.7)		51 (28.8)	17 (41.4)	

Gender differences were assessed using Chi-squared and t-tests, respectively, both in patients with and without depressive symptoms

*P* values in bold are significant at *p* < 0.05

*CDS* Cardiac depression scale, *HS* High school, *MI* Myocardial infarction, *CHD* Coronary heart disease, *CATH* Catheterization, *CABG* Coronary artery bypass graft, *PHQ-15* Patient health questionnair-15, *QoL* Quality of life, *SF-12* Short-form 12 health survey, *PCS* Physical component summary, *SD* Standard deviation, *PTSD* Post-traumatic stress disorder, *PTSD-PCL-S* Post-traumatic stress disorder checklist, *RS-14* Resilience scale-14, *SISE* Single-item self-esteem scale, *ESSI* ENRICHD social support instrument, *MCS* Mental component summary, *BMI* Body mass index

^a^Independent *t* test

Differences among women and men with depressive symptoms in terms of clinical factors were also found. Women with depressive symptoms were more likely to have a severe cardiac disease (i.e. heart failure, mitral or aortic valve stenosis) compared to men with depressive symptoms who had mostly CHD or a MI diagnosis, more likely to report a previous cardiac diagnosis, undergo a CATH/complex procedure (i.e. valve replacement), have two or more comorbid conditions (i.e. diabetes, hypertension, the combination of both), take three to four medications, have higher somatic symptoms and to score lower on the SF-12-PCS for quality of life.

Bivariate comparisons of psychosocial factors between depressed men and women revealed that depressed women were more likely to have higher levels of PTSD symptoms, lower social support, lower resilience, lower self-esteem and a lower score on the SF-12-MCS for quality of life than their male counterparts.

Regarding lifestyle factors, women with depressive symptoms were less likely to be smokers and have a high fat intake, while more likely to be physically inactive and to be obese than men with depressive symptoms.

In the sample without depressive symptoms, the pattern of gender differences was similar, but with the small sample of women without depression (*n* = 41), significant differences were observed less frequently.

**Fig. 1:**
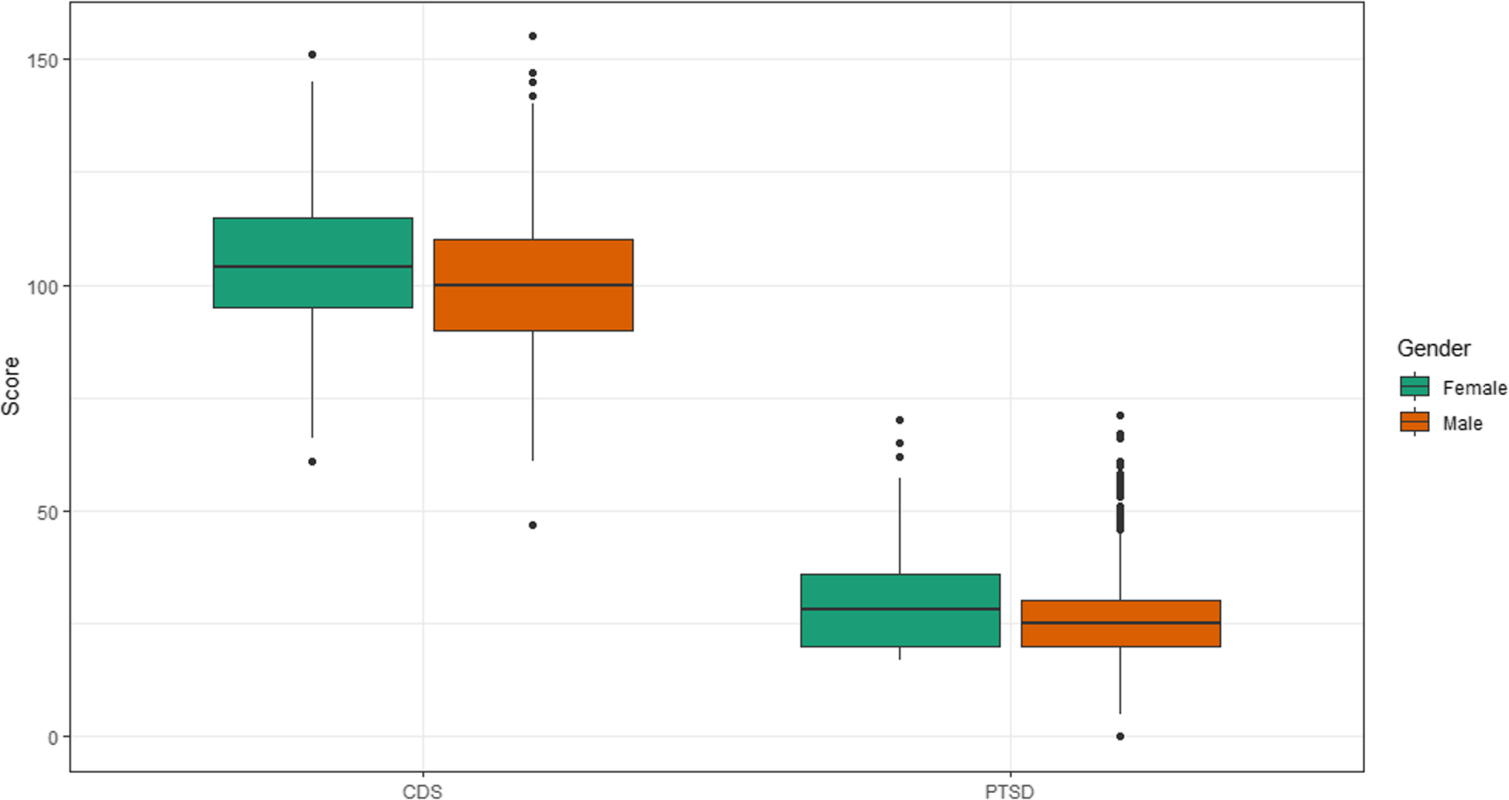
Distribution of scores for symptoms of depression (CDS score) and PTSD score, by gender. *Note*. CDS=Cardiac Depression Scale (Possible score range: 26–182; Actual score range: 47–155); PTSD = Post-traumatic Stress Disorder (Possible score range: 0–85; Actual score range: 0–71)

The presence of depressive symptoms as defined by a CDS score ≥ 90 among the total sample was 78.7%. The proportion of severe depressive symptoms (CDS > 100) was higher in women compared to men (61.4% versus 50.7%, *P =* 0.003), while the proportion of women without depressive symptoms was 15.1% versus 23.6% for men (Table 2). Comparison of depressive and PTSD symptoms assessed as continuous variables (means of CDS and PTSD scores) by gender revealed that women had significantly higher CDS (104.6 ± 15.9; *t* = 4.1; *P* < 0.001) and PTSD scores (29.4 ± 10.6; *t* = 4.4; *P* < 0.001) (Fig. 1).

**Table 2:** Frequency of levels of severity of depressive symptoms, by gender

CDS score	Total Population (*N* = 1022)	Men (*N* = 750)	Women (*N* = 272)	*P* value
< 90: no depression	218 (21.3)	177 (23.6)	41 (15.1)	0.003
90–100: mild to moderate depression	257 (25.2)	193 (25.7)	64 (23.5)	
> 100: severe depression	547 (53.5)	380 (50.7)	167 (61.4)	

*CDS* Cardiac depression scale

### Direct and indirect effects of gender on depression (CDS score)

**Fig. 2:**
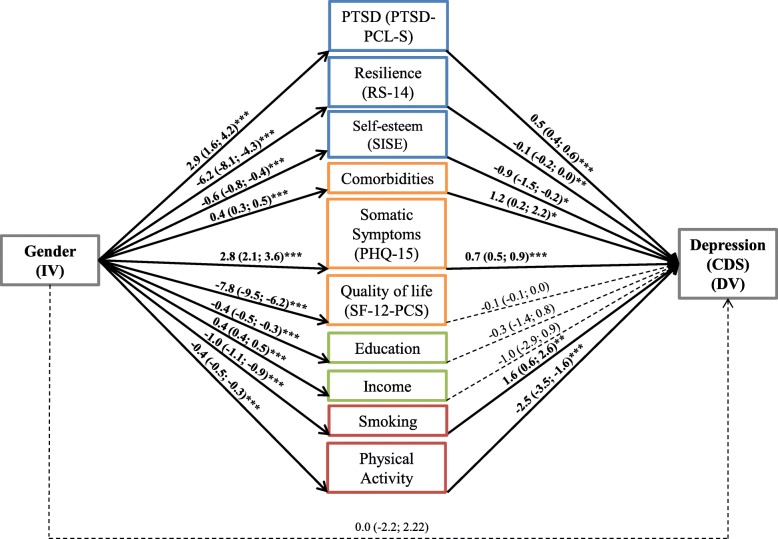
SEM of the direct and indirect effects of gender on depression (CDS score), (*N* = 1022). *Note.* The pathways represented by arrows correspond to direct and indirect effects of gender on depression (CDS score). Solid arrows (in bold) represent statistically significant associations between variables and dashed arrows represent non-significant associations. Each arrow is labeled with the respective effect estimate and its 95%-confidence interval. The effect magnitudes on the arrows are not comparable to each other because the mediators have different scale ranges.Gender was coded as 0 = male, 1 = female; IV = independent variable, DV = dependent variable, CDS=Cardiac Depression Scale, PTSD-PCL-S=Post-traumatic Stress Disorder Checklist, RS-14 = Resilience Scale-14, SISE = Single-Item Self-Esteem Scale, SF-12-PCS = Short-form 12 Health Survey-Physical Component Summary, PHQ-15 = the Patient Health Questionnaire-15; *p < 0.05, **p < 0.01, ***p < 0.001

The pooled SEM of the direct effect of gender on depression (CDS score) and the indirect effects when mediators were added was conducted for the overall sample. Figure 2 illustrates the pathways between gender and depression with the tested mediators. Parameters reported in the figure include coefficients and confidence intervals (CI). They demonstrate the associations of gender with each potential mediating factor and between each potential mediating factor and outcome variable, after adjusting for the effect of gender. Results from the SEM showed both positive and negative mediators, where positive mediators mediating the gender difference on depression are associated both with being female and higher levels of depression. In the pooled SEM of gender and depression, there was no direct effect of gender on CDS score (coefficient: 0.0, 95% CI: − 2.2-2.2, *p* = 1.0). There were significant direct effects of gender on the mediators as shown in Fig. 2. On the one hand, female gender was positively associated with higher PTSD symptoms, comorbidities, somatic symptoms and income. On the other hand, female gender was associated with lower resilience, self-esteem, quality of life, education, less smoking and lower physical activity. Negative indirect effects of gender on depression were observed through resilience, self-esteem, quality of life, (SF-12-PCS), education, income and physical activity, whereas positive indirect effects of gender on depression were observed through PTSD, comorbidities, somatic symptoms, and smoking. Quality of life, education, and income were not significant mediators for the indirect effect of gender on depression. No evidence of moderation was found for the direct effect of gender on CDS score, the effects of gender on mediators and the effects of mediators on CDS score.

There was no indication of confounding or moderation by age. In the pooled model, age was not significant when a direct effect of age on CDS score was added to the model. There were also no significant differences between corresponding effect estimates after SEM’s were conducted separately for the two age groups < 59 years and ≥ 59 years. These results are presented in Additional file 1: Figure S1a and b.

The SEM conducted to assess whether resilience could also be a mediator and/or moderator between PTSD and depression (CDS score) revealed a significant direct association between PTSD and depression and a small positive moderating effect of resilience on this association was observed. There were negative yet significant associations between PTSD and resilience and between resilience and depression (see Additional file 2: Figure S2). Thus, in this SEM, resilience appeared both as a mediator of the total effect of PTSD on depression and a moderator of the respective direct effect.

**Fig. 3:**
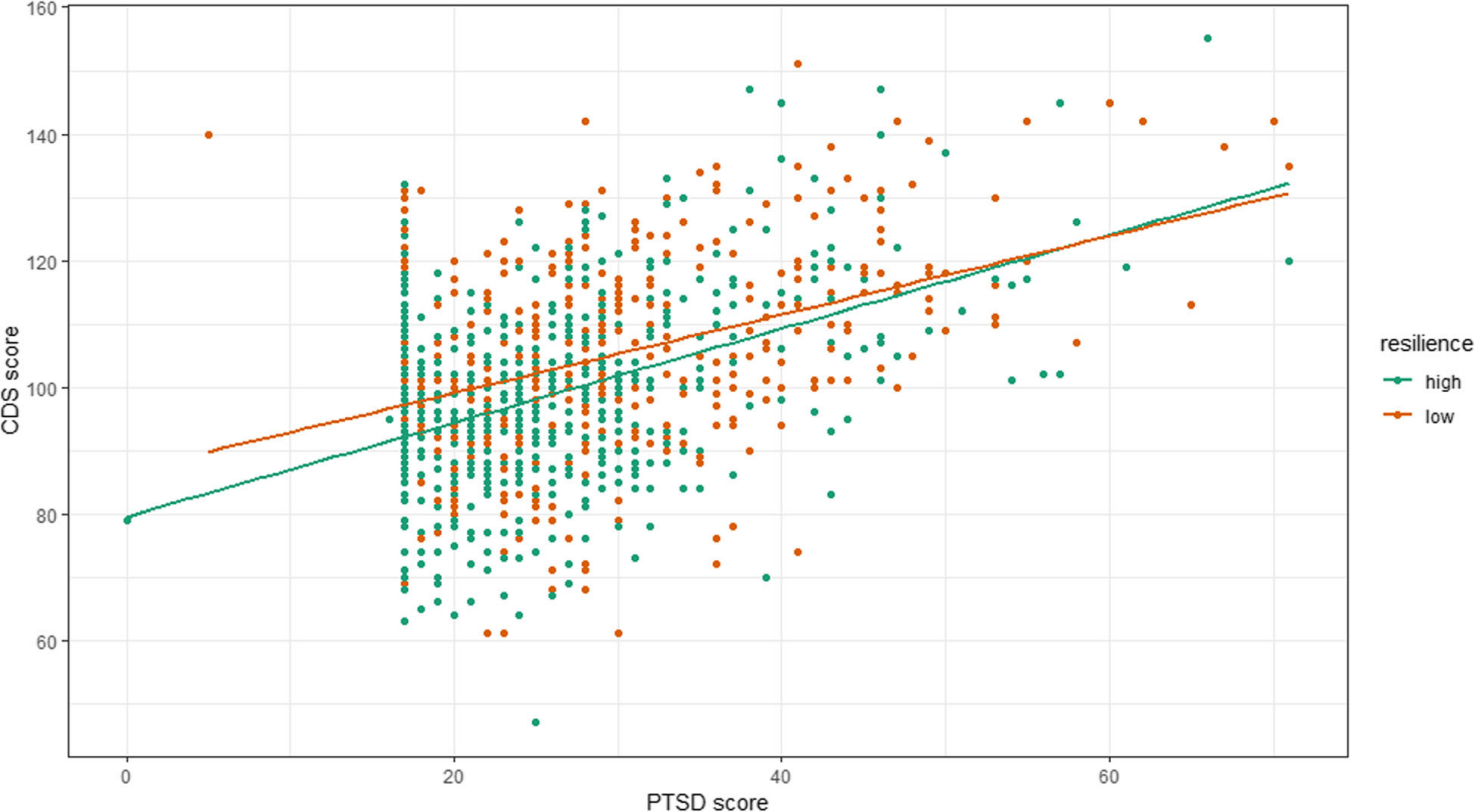
Association of PTSD and CDS stratified by level of resilience. *Note.* CDS = Cardiac Depression Scale; PTSD = Post-traumatic stress disorder

Figure 3 illustrates the interaction between PTSD and resilience, in which the difference in predicted depression (CDS score) between those with low and high resilience decreases slightly with increasing PTSD symptoms. This suggests that resilience shows a clearer protective role at lower levels of PTSD, becoming less protective at higher levels of PTSD.

No mediation by smoking of the association between PTSD and depression (CDS score) was found (data not shown).

## Discussion

In our sample of cardiac patients, women with CVD have a higher prevalence of severe depressive symptoms (61.4%) compared to men (50.7%). The association of gender with depression was entirely explained by a number of mediators. The strongest positive mediator was PTSD, which was more prevalent in women and positively associated with depression. While resilience itself was a negative mediator of the gender-depression association, it mediated and moderated the PTSD-depression (CDS score) association. Besides mediation by psychosocial parameters, lifestyle factors such as physical activity and smoking also explained part of the gender-depression association.

Our findings that women with CVD were more likely to experience depressive symptoms and at higher levels than men with CVD were consistent with other studies [50–52]. This finding is also consistent with the respective sex difference observed in the general population [9].

Previously, there has been evidence on gender being linked to PTSD and comorbid depression as well as PTSD and associated outcomes in cardiac patients. However, to our knowledge, this is the first study to assess direct and indirect effects of gender on depression among a cardiac population, with a particular focus on PTSD as a mediator. Previous studies have found that women are at higher risk of comorbid PTSD and depression symptoms compared to men [53, 54], similar to our findings which showed a direct significant association of female gender with PTSD and a significant indirect effect of gender on depression mediated by PTSD. Reasons for these gender differences could be attributed to the different types of traumatic or stressful situations which men and women are exposed to, different responses to trauma, and different symptom-related responses, all of which may affect the course and severity of symptoms [55]. In addition, higher rates of PTSD and depression among women could be due to women’s lower social position which can lead to greater exposure to trauma and stressors compared to men [56]. This could particularly be true among our sample and in the cultural context of Palestine, where women have much lower social positions compared to men. In the present study, women had more severe diagnoses, comorbidities and complicated treatment procedures along with other negative daily stressors, suggesting that women were more vulnerable to traumatic stress which arises from both PTSD and depression and their comorbidity. Dao et al., found increased mortality among comorbid PTSD and depression patients after CABG to be linked to female gender [49]. These results have implications for early assessment of these mental health disorders, as their comorbidity is becoming more frequently observed and prevalent in cardiac patients.

Our findings also reveal that low resilience is associated with being female and higher depressive symptoms among our cardiac sample. This finding supports the results of a study by Carvalho et al. [57], which found that among a CVD sample, lower scores of resilience were associated with depressive symptoms and with female gender, compared to men. This finding could indicate that men tend to have more motivation to focus on problem solving coping strategies, whereas women tend to use emotion-focused coping strategies [11, 58]. Additionally, another study which assessed for depression using the CDS found a strong association between low resilience and depression (CDS > 95) among CVD patients [59]. In our further analysis assessing mediation of resilience between PTSD and depression (CDS score), we found a significant effect for the interaction by resilience of the association between PTSD and depression (CDS score). We also found the difference in CDS score between those with low and high resilience decreased with increasing PTSD symptoms, potentially meaning that higher levels of PTSD partly override the protective effects of resilience. These findings suggest the need for intervention targets such as group cardiac rehabilitation and interventions to improve coping with adversities, which could ultimately reduce the risk of depression and strengthen resilience among cardiac patients. Furthermore, promoting physical activity among CVD patients is recommended as resilience also has been linked to exercise [60] and has been known to reduce the risk of depression among resilient patients with CVD [59].

Higher somatic symptoms were also associated with being female and higher depression levels in this study, consistent with previous reports which found females with major depression showed higher rates of somatic symptoms compared to men [61, 62]. A study conducted in Spain, which also used the PHQ-15 to assess for somatic symptoms, found women reported higher total scores than men and were more concerned in 8 out of 15 items of the PHQ-15 [63]. Nonetheless, these findings should be interpreted with caution since somatic symptom scales vary in different studies.

Our results also reveal that physical inactivity is associated with being female and higher depressive symptoms among our cardiac sample. It has been previously reported that female cardiac patients frequently engage in less exercise compared with their male counterparts, despite the increasing evidence to support the beneficial role of exercise among cardiac patients [64]. Physical inactivity has been associated with depression and has been shown to mediate the relationship between depression and cardiovascular events and mortality [65, 66]. A recent systematic review found that depression could lead to a sedentary lifestyle and lower levels of physical activity [67]. These data along with our findings suggest that there is need for exercise programs for CVD patients, especially women and those with depression.

Furthermore, our findings shed light on gender differences for socioeconomic factors among men and women with both CVD and depressive symptoms. In the present study, and similar to other studies, women were more likely to be not married, unemployed, and less educated [68, 69], compared to men with CVD and comorbid depressive symptoms. These findings are supported by previous studies on CHD patients that female gender is linked to lower education, which increases the risk of onset of CHD [70], whereas employment and higher education play a protective role against depressive symptoms [71]. Those with high socioeconomic status have more self-esteem, more resources and social support, all of which reduce the stress that comes with depression compared to those with low socioeconomic status, whom have less access to social and personal resources, which increases stress levels. However, occupation and marital status were not significant in the final SEM and thus were not mediating effects of gender on depression (CDS score).

Furthermore, in our sample, women had more coexisting illnesses (i.e. diabetes and hypertension) compared with men with cardiac disease, a finding consistent with other studies [68, 69, 72]. There were no major differences in rates of angina as an initial cardiac diagnosis between men and women, which is inconsistent with results in other studies, which found higher angina rates among women compared with men [73, 74].

### Strengths

This study had several strengths. First, the high participation rate decreased the likelihood of selection and participation bias. Second, our sample of women was relatively large, compared to other studies, which provided sufficient statistical power for testing gender differences. Third, the CDS used in the study is the only psychometric scale suitable for the comparative depression assessment in heart disease patients, subjected to different interventions and has excellent properties for the diagnosis of severe depression, a score > 100 having a sensitivity of 88% and a specificity of 84%. Finally, the findings of our study are likely generalizable as our sample was recruited from four main hospitals of Nablus, Palestine, which provide cardiac care to a large percentage of individuals in the area.

### Limitations

The limitations of our study are due to several sources. First, depression was not formally diagnosed by a psychological interview and was rather assessed using the CDS, thus we cannot conclude that our sample had clinical depression. However, the identification of certain mediating factors of gender on depression among cardiac patients can serve as a basis for developing the best intervention targeting depression among women and men with cardiac disease. Second, some patients were recruited at the time of admission and for the most part before receiving their intervention or diagnosis, and thus were under stress and anxiety. As some of the anxiety observed may have been transient, the level of depression and anxiety could have been overestimated. Finally, due to the cross-sectional design of the study the direction of associations cannot be derived with certainty and causality cannot be inferred.

## Conclusion

This study demonstrated that, similar to the general population, the prevalence of severe depression is greater in female patients with cardiac disease compared to male patients. Overall, our findings provide new information documenting mediating factors, in particular PTSD, of the association between gender and depression among cardiac patients. These findings emphasize the need to devote more attention to identify and address potential mediating factors that could account for gender differences in depression. Providing supportive programs for female patients with CVD to improve their psychosocial well-being in cardiac rehabilitation seems of particular importance in conflict-prone areas, like Palestine.

## Additional files

Additional file 1: **Figure S1a, 1b.** SEM of the direct and indirect effects of gender on depression (CDS score) for age groups < 59 (*N = 500*) and ≥ 59 (*N = 522*). (DOCX 140 kb)

Additional file 2: **Figure S2.** SEM of association between resilience, PTSD and depression (CDS score). (PNG 9 kb)

## Data Availability

The datasets used and/or analyzed during the current study are available from the corresponding author on reasonable request.

## Abbreviations

BMI: Body mass index
CABG: Coronary artery bypass graft
CATH: Catheterization
CDS: Cardiac Depression Scale
CHD: Coronary heart disease
CI: Confidence interval
CVD: Cardiovascular disease
DASS-42: Depression Anxiety Stress Scale-42
DV: Dependent variable
EKNZ: Ethics Committee of Nordwest-und Zentral Schweiz
ENRICHD: Enhancing Recovery in Coronary Heart
ESSI: ENRICHD Social Support Instrument
HF: Heart failure
HS: High school
IRB: Institutional Review Board
IV: Independent variable
MCS: Mental Component Summary
MDD: Major depressive disorder
MI: Myocardial infarction
MOS: Medical Outcomes Survey
oPt: Occupied Palestinian territory
PCS: Physical Component Summary
PHQ-15: Patient Health Questionnaire-15
PTSD: Post-traumatic stress disorder
PTSD-PCL-S: Post-Traumatic Stress Disorder Checklist
QOL: Quality of life
RS-14: Resilience Scale-14
SD: Standard deviation
SE: Self-esteem
SEM: Structural equation model
SF-12: The 12-item Short Form Health Survey
