# Supplementary material for: Mediators of gender effects on depression among cardiovascular disease patients in Palestine
Source: BMC Psychiatry. 2019 Sep 12;19:284. doi: 10.1186/s12888-019-2267-4 (PMC6739957; doi:10.1186/s12888-019-2267-4)
Supplement: Supplementary file 1 — Figure S1a, 1b. SEM of the direct and indirect effects of gender on depression (CDS score) for age groups < 59 (N = 500) and ≥ 59 (N = 522). (DOCX 140 kb) [file 12888_2019_2267_MOESM1_ESM.docx]

**Figure S1a, 1b** SEM of the direct and indirect effects of gender on depression (CDS score) for age groups <59 (*N=500*) and ≥59 (*N=522*).

*Age group <59*


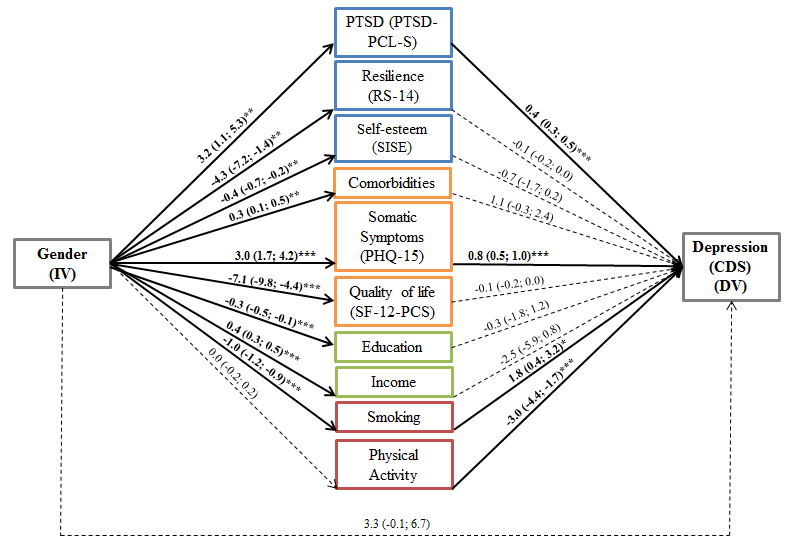
*Note.* The pathways represented by arrows correspond to direct and indirect effects of gender on depression (CDS score). Solid arrows (in bold) represent statistically significant associations between variables and dashed arrows represent non-significant associations. Each arrow is labeled with the respective effect estimate and its 95%-confidence interval. The effect magnitudes on the arrows are not comparable to each other because the mediators have different scale ranges.

Gender was coded as 0=male, 1=female; IV=independent variable, DV= dependent variable, CDS=Cardiac Depression Scale, PTSD-PCL-S=Post-Traumatic Stress Disorder Checklist, RS-14=Resilience Scale-14, SISE= Single-Item Self-Esteem Scale, SF-12-PCS= Short-form 12 Health Survey-Physical Component Summary, PHQ-15= the Patient Health Questionnaire-15; *p<0.05, **p<0.01, ***p<0.001


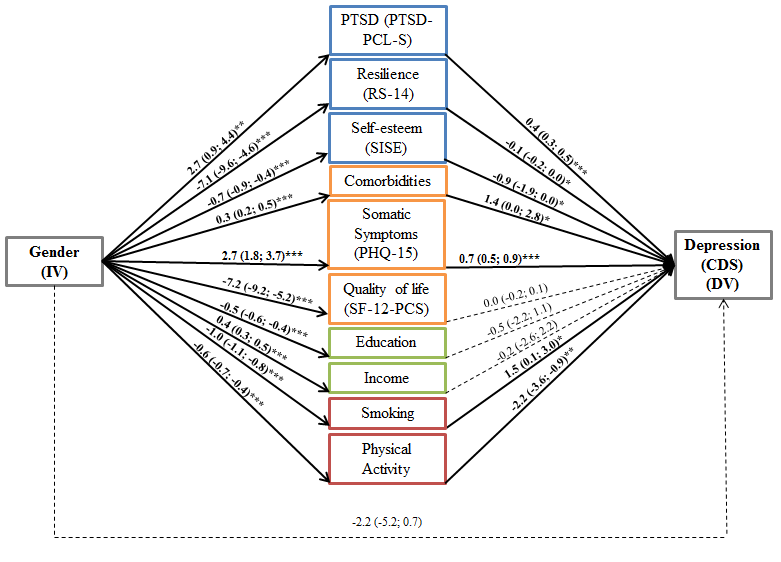
*Age group ≥59*

*Note.* The pathways represented by arrows correspond to direct and indirect effects of gender on depression (CDS score). Solid arrows (in bold) represent statistically significant associations between variables and dashed arrows represent non-significant associations. Each arrow is labeled with the respective effect estimate and its 95%-confidence interval. The effect magnitudes on the arrows are not comparable to each other because the mediators have different scale ranges.

Gender was coded as 0=male, 1=female; IV=independent variable, DV= dependent variable, CDS=Cardiac Depression Scale, PTSD-PCL-S=Post-Traumatic Stress Disorder Checklist, RS-14=Resilience Scale-14, SISE= Single-Item Self-Esteem Scale, SF-12-PCS= Short-form 12 Health Survey-Physical Component Summary, PHQ-15= the Patient Health Questionnaire-15; *p<0.05, **p<0.01, ***p<0.001
