# Supplementary figures and images for: Mediators of gender effects on depression among cardiovascular disease patients in Palestine
Source: BMC Psychiatry. 2019 Sep 12;19:284. doi: 10.1186/s12888-019-2267-4 (PMC6739957; doi:10.1186/s12888-019-2267-4)

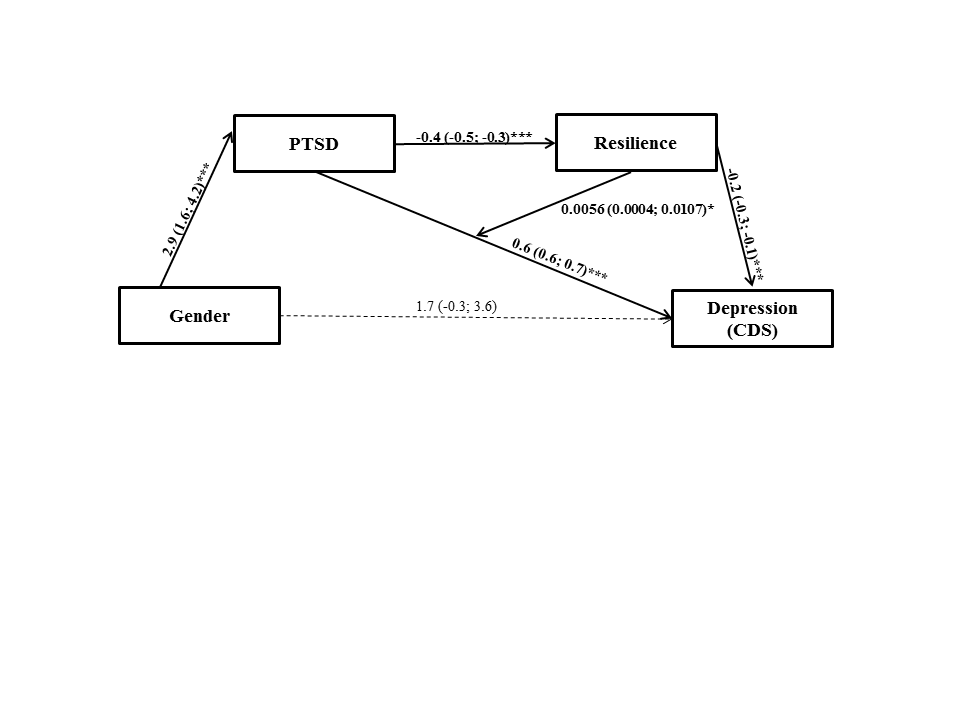

Supplement: Supplementary file 2 — Figure S2. SEM of association between resilience, PTSD and depression (CDS score). (PNG 9 kb) [file 12888_2019_2267_MOESM2_ESM.png]
